# The volcano lesions may reflect a pathological phenomenon in metastatic melanoma of the gastrointestinal tract

**DOI:** 10.1016/j.igie.2026.03.008

**Published:** 2026-07-29

**Authors:** Akira Hokama

**Affiliations:** Department of Medical Checkup, Naha City Hospital, Naha, Japan

To the Editor:

I read with great interest the article by Ohri et al,^1^ which presents impressive images of metastatic melanoma with the “volcano” lesions. I congratulate the authors on emphasizing the importance of this characteristic endoscopic feature for detecting metastasis. I would like to comment on the possible pathological basis of this appearance. The endoscopic appearance of an elevated, ulcerated volcano is a classic finding called a “target sign” lesion or “bull's-eye” lesion on barium follow-through radiological imaging.^2^^,^^3^ Melanoma cells exhibit high angiogenic activity, which enables aggressive metastatic potential and drug resistance.^4^ A pathological study of metastatic melanoma revealed the presence of melanoma cells within channels lined by lymphatic endothelium in the submucosa and serosa of the gastrointestinal tract.^5^ The study also showed that nodules of carcinoma cells arise in the submucosa, stretching the muscularis mucosae and mucosa over their convex summits. Ulceration at the tip may be caused by necrosis. These features may form submucosal tumorlike masses with ulcerated centers. Additionally, metastatic lesions are often amelanotic^6^^,^^7^; therefore, immunostaining methods, as demonstrated by the authors, are necessary. In conclusion, recent advances in immune checkpoint inhibitors have revolutionized melanoma therapy. The excellent images of Ohri et al^1^ highlight the importance of early detection of metastatic melanoma and its clinical implications for metastatic pathogenesis.

## Disclosure

The author disclosed no financial relationships.
